# Phenotypic Covariance of Longevity, Immunity and Stress Resistance in the *Caenorhabditis* Nematodes

**DOI:** 10.1371/journal.pone.0009978

**Published:** 2010-04-01

**Authors:** Francis R. G. Amrit, Claudia M. L. Boehnisch, Robin C. May

**Affiliations:** School of Biosciences, College of Life and Environmental Sciences, University of Birmingham, Birmingham, West Midlands, United Kingdom; Centre for Genomic Regulation, Spain

## Abstract

**Background:**

Ageing, immunity and stresstolerance are inherent characteristics of all organisms. In animals, these traits are regulated, at least in part, by forkhead transcription factors in response to upstream signals from the Insulin/Insulin–like growth factor signalling (IIS) pathway. In the nematode Caenorhabditis elegans, these phenotypes are molecularly linked such that activation of the forkhead transcription factor DAF-16 both extends lifespan and simultaneously increases immunity and stress resistance. It is known that lifespan varies significantly among the Caenorhabditis species but, although DAF-16 signalling is highly conserved, it is unclear whether this phenotypic linkage occurs in other species. Here we investigate this phenotypic covariance by comparing longevity, stress resistance and immunity in four Caenorhabditis species.

**Methodology/Principal Findings:**

We show using phenotypic analysis of DAF-16 influenced phenotypes that among four closely related Caenorhabditis nematodes, the gonochoristic species (Caenorhabditis remanei and Caenorhabditis brenneri) have diverged significantly with a longer lifespan, improved stress resistance and higher immunity than the hermaphroditic species (C. elegans and Caenorhabditis briggsae). Interestingly, we also observe significant differences in expression levels between the daf-16 homologues in these species using Real-Time PCR, which positively correlate with the observed phenotypes. Finally, we provide additional evidence in support of a role for DAF-16 in regulating phenotypic coupling by using a combination of wildtype isolates, constitutively active daf-16 mutants and bioinformatic analysis.

**Conclusions:**

The gonochoristic species display a significantly longer lifespan (p<0.0001) and more robust immune and stress response (p<0.0001, thermal stress; p<0.01, heavy metal stress; p<0.0001, pathogenic stress) than the hermaphroditic species. Our data suggests that divergence in DAF-16 mediated phenotypes may underlie many of the differences observed between these four species of Caenorhabditis nematodes. These findings are further supported by the correlative higher daf-16 expression levels among the gonochoristic species and significantly higher lifespan, immunity and stress tolerance in the constitutively active daf-16 hermaphroditic mutants.

## Introduction

Longevity is a phenomenon shared by all living organisms but which varies hugely across species and between different sexes of the same species. Several evolutionary theories have been postulated to explain this phenomenon, but the underlying biological regulators of longevity remained largely unknown until pioneering genetic studies using the roundworm *Caenorhabditis elegans* identified the first gene with a substantial role in determining lifespan [1], [2], [3].

Given that post-reproductive survival cannot evolve under direct selection, diapause (the entry into a semi-dormant state with low metabolic turnover) is generally perceived as being a by-product of a survival strategy triggered by the organism to outlive harsh conditions so that, upon encountering a suitable environment, reproduction can be resumed [4]. One such strategy employed by the *Caenorhabditis* nematodes is to enter into a temporary, developmentally arrested dauer stage. [5], [6], [7]. In *C. elegans*, this phenomenon is regulated by the IIS (Insulin/Insulin–like growth factor (IGF) signalling) pathway, which consists of a transmembrane protein DAF-2 [8], several intracellular kinases and the DAF-16 transcription factor [9]. When inactivated, this pathway not only extends lifespan but also regulates resistance to pathogens and abiotic stresses [10], [11], [12]. Mutations in this pathway, such as inhibitory mutations in *age-1* (a homologue of the mammalian phosphatidylinositol 3-OH kinase) or *daf-2* (a homologue of the mammalian insulin receptor) result in the relocalization of the transcription factor DAF-16 into the nucleus where it regulates a plethora of downstream genes [2], [13], [14], [15], [16].

The IIS pathway is highly conserved in organisms ranging from yeast to humans and, in many cases, appears to retain its dual role as a major effector of immunity and longevity [17], [18], [19], [20]. Studies in *C. elegans* have explored this coupling relationship between the *daf-16* determined phenotypes of longevity, immunity and stress tolerance to a great extent, but little is known about the corresponding phenotypes in other nematode species.

Here we provide experimental data to address this question by undertaking a comprehensive analysis of immunity, stress response and longevity phenotypes in several representative isolates of four nematode species within the same genus. We demonstrate that, within this group of closely related animals, there exists a high divergence with regards to traits such as lifespan and stress tolerance and, intriguingly, in the expression of *daf-16*. Furthermore, we investigated conservation in the DAF-16 downstream regulon (target genes) by surveying the three available *Caenorhabditis* genomes (*C. elegans, C. briggsae* and *C. remanei*) for genes containing the known consensus sites for DAF-16. Based on orthologous sets of genes containing the consensus sites, we asked whether certain biological processes are more prevalent in one species than in others (divergent targets) and which processes are shared between all three species. We also tested for adaptive sequence evolution along the IIS pathway in these species. Finally, we use classical genetics to constitutively activate the DAF-16 pathway in two *Caenorhabditis* species in order to experimentally identify both conserved and divergent downstream phenotypes.

## Results

### Gonochoristic species are longer lived than the hermaphroditic species and show higher levels of *daf-16* expression

**Figure 1:**
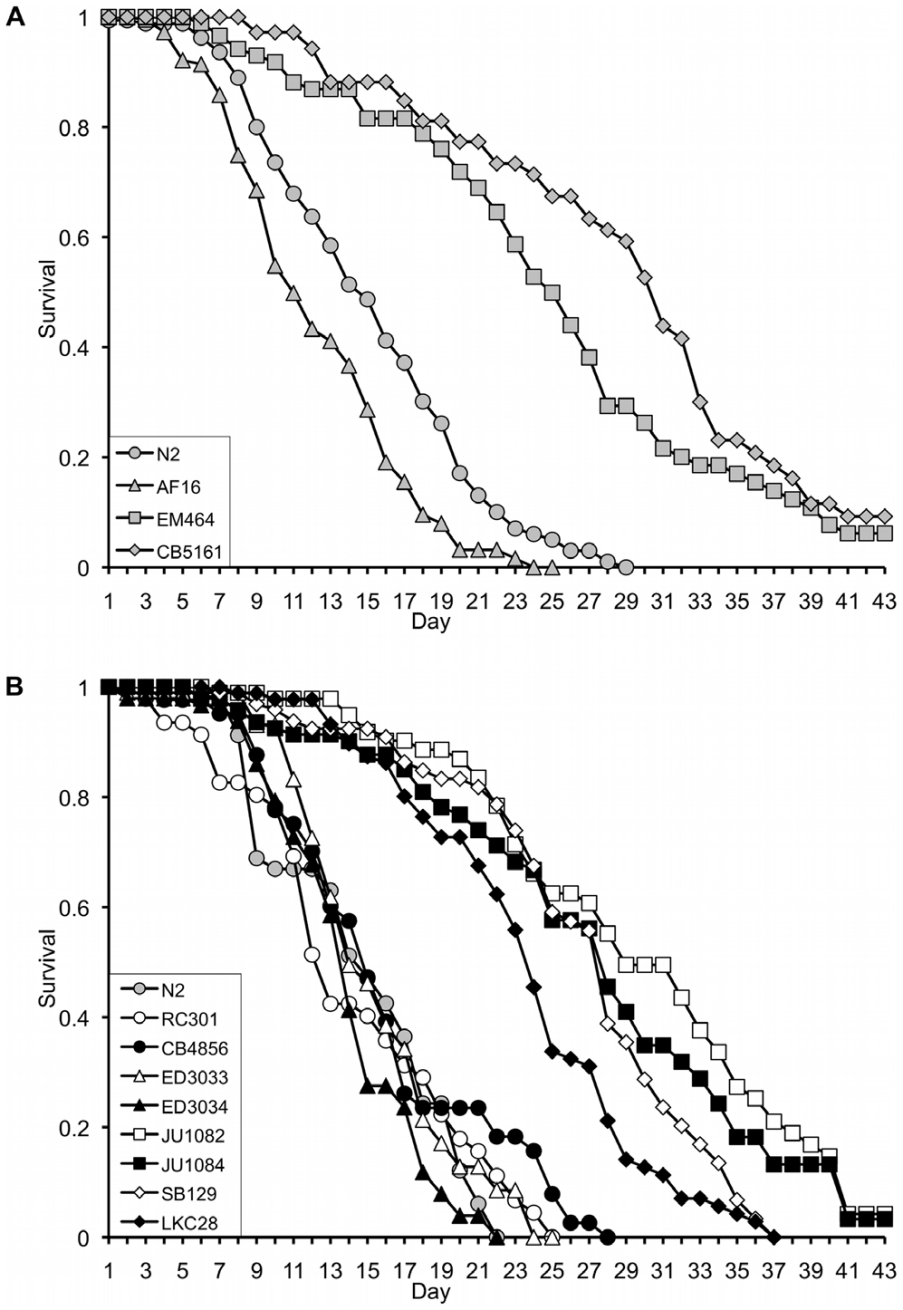
Lifespan analysis. (a) Hermaphrodite (N2, AF16) or female (EM464, CB5161) animals at the fourth larval stage (L4) were transferred onto plates pre-seeded with OP50 and monitored for survival over fifty days. Whilst hermaphrodite animals show 100% lethality over this period, survival is significantly higher for both gonochoristic species (p<0.0001, Table S1), with more than 50% of animals surviving longer than twenty days. (b) This effect is conserved across multiple wildtype isolates of each species.

We and others have previously demonstrated that different *Caenorhabditis* species exhibit significantly different lifespans [21], [22]. Since different laboratory isolates can exhibit variation in lifespan [23], we conducted parallel longevity assays on our isolates of *C. elegans* N2, *C. briggsae* AF16, *C. remanei* EM464 and *C. brenneri* CB5161. As previously reported [22], the two gonochoristic species (*C. remanei* and *C. brenneri*) exhibit a significantly (p<0.0001; see Table S1 for all p-values) longer lifespan than both hermaphroditic species (*C. elegans* and *C. briggsae*) (Figure 1a). Additional testing confirmed that this trend was highly conserved across multiple wildtype isolates of each species (Figure 1b, Table S2), as previously reported [22]. The testing of several hermaphroditic wildtype isolates also ruled out the possibility that these observations were due to the fixation of novel mutations under the force of genetic drift in our laboratory *C.elegans* (N2) line.

**Figure 2:**
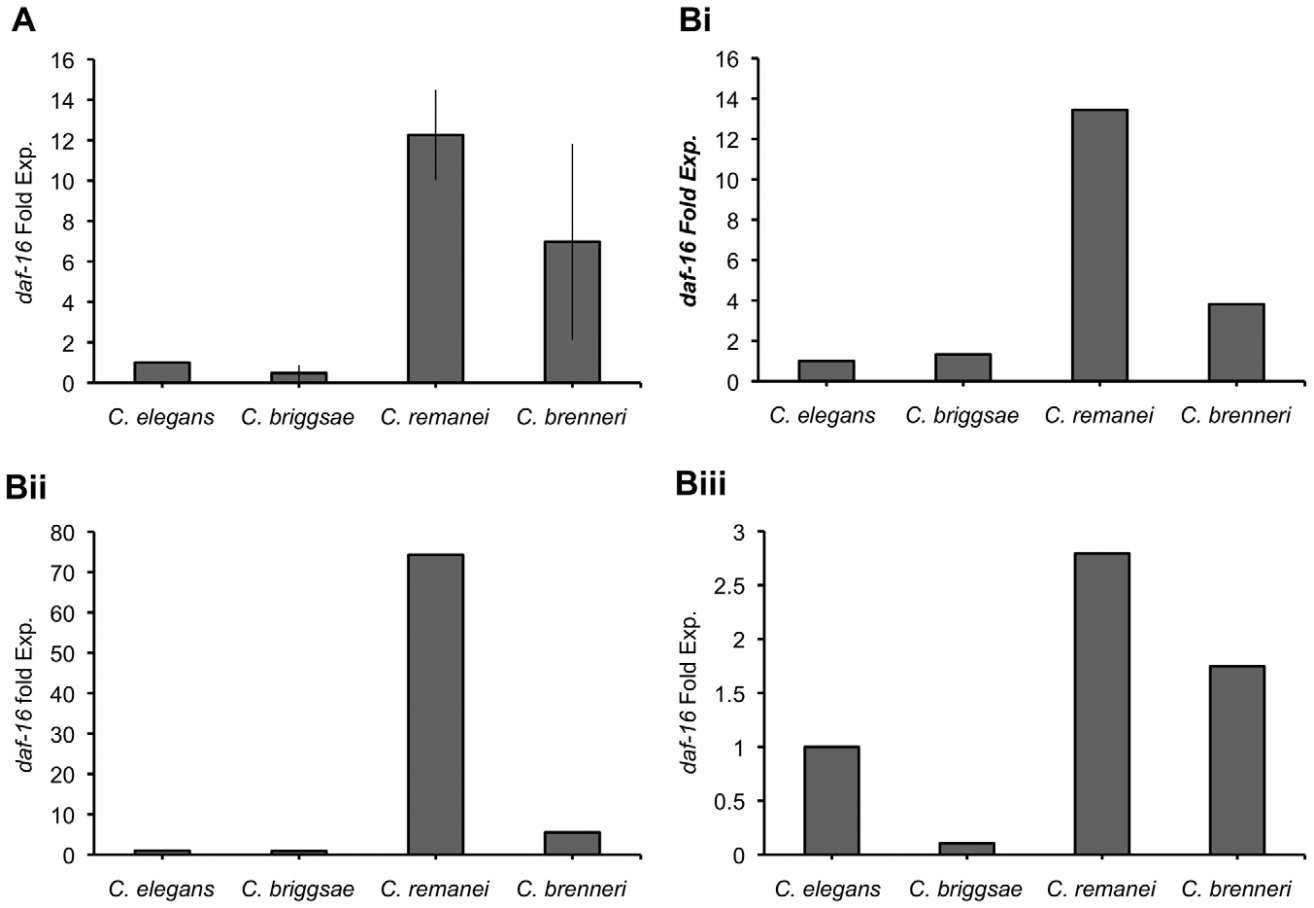
Quantitation of *daf-16* gene expression. (a) mRNA from a mixed population of hermaphroditic (N2, AF16) and gonochoristic (EM464, CB5161) animals was extracted and *daf-16* gene expression was quantified relative to the housekeeping gene *gpd-3*. Data represent the mean of three experiments, error bars show standard deviation. (b) *daf-16* gene expression for the same species but measured at various stages of development that include L2-L3 stage (bi), L4 stage (bii) and the adult stage (biii).

Given the evolutionary conservation and critical role played by DAF-16 in regulating lifespan in *C. elegans*, we quantified *daf-16* mRNA levels in both mixed populations (nematodes at various stages of development) and staged populations (L2-L3, L4 and adult stages) of all strains of the four *Caenorhabditis* species. Whilst *C. briggsae* showed *daf-16* levels similar to those in *C. elegans*, *daf-16* expression in the two gonochoristic species was between seven (*C. brenneri*) and twelve (*C. remanei*) fold higher than *C. elegans* in the mixed populations (Figure 2a). Higher *daf-16* expression levels among the gonochoristic species was also observed throughout development in the staged populations (Figure 2bi, 2bii & 2biii) with the difference being most prominent in the L4 stage (Figure 2bii). Thus higher levels of *daf-16* expression seem to positively correlate with longer lifespan.

We also tested for expression levels of *daf-16* in *C.elegans* males using two independent reference genes (Table S3) and found no significant transcriptional difference in comparison to *C. elegans* hermaphrodites, indicating that the absence or presence of males in a population has no effect with regards to *daf-16* expression levels. We note that the presence of both males and females within the gonochoristic population could mean that enhanced *daf-16* expression may be restricted to one or other gender, but given that both male and female animals in the gonochoristic species are longer lived than either gender of *C. elegans* or *C. briggsae* [21], we regard it as more likely that *daf-16* is highly expressed in both genders of the gonochoristic species.

### Long-lived species are more resistant to abiotic stress than shorter-lived species

**Figure 3:**
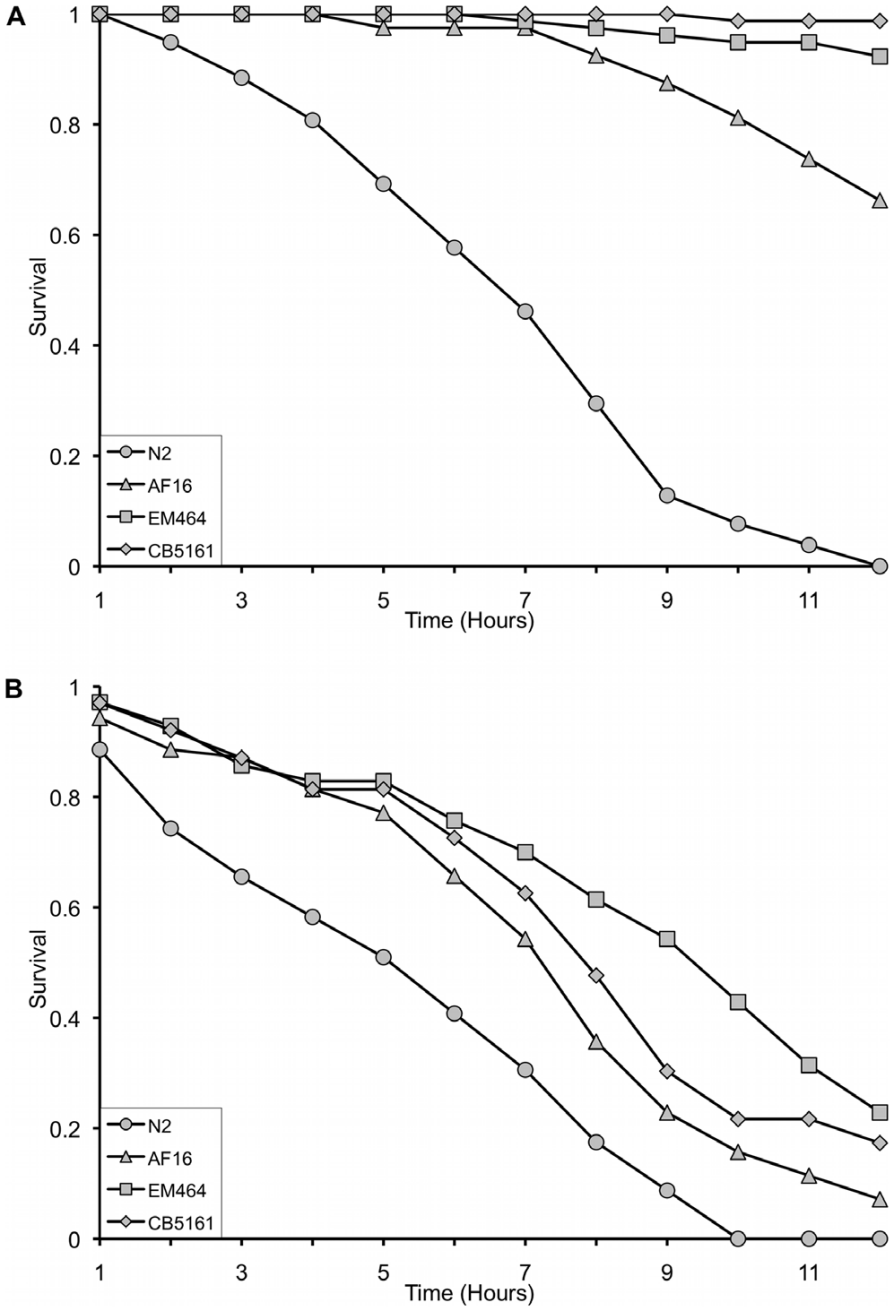
Survival analysis following exposure to abiotic stress (heat and heavy metal). L4 Hermaphrodite (N2, AF16) or female (EM464, CB5161) animals were monitored for survival (a) at 37°C or (b) during exposure to 7 mM copper chloride. *C. elegans* and *C. briggsae* show significantly higher susceptibility to both high temperature (p<0.0001, Table S1) and heavy metal toxicity (p<0.01, Table S1).

In *C. elegans*, DAF-16 activity substantially increases survival following exposure to high temperature or heavy metals [10], [24]. To test whether the observed higher levels of *daf-16* in the gonochoristic species also correlate with better survival to abiotic stress, we exposed multiple isolates of all four species to prolonged high temperature of 37°C (Figure 3a) or toxic heavy metals such as CuCl_2_ (Figure 3b, Figure S2). In both cases, the gonochoristic species showed significantly (Table S1) higher survival than either hermaphroditic species. The correlation of these phenotypes with *daf-16* expression levels, together with prior knowledge from studies in *C. elegans,* suggests that higher DAF-16 levels could potentially be driving both increased lifespan and increased resistance to abiotic stress.

### Longer-lived species in general are more resistant to biotic stress factors than the shorter-lived species

**Figure 4:**
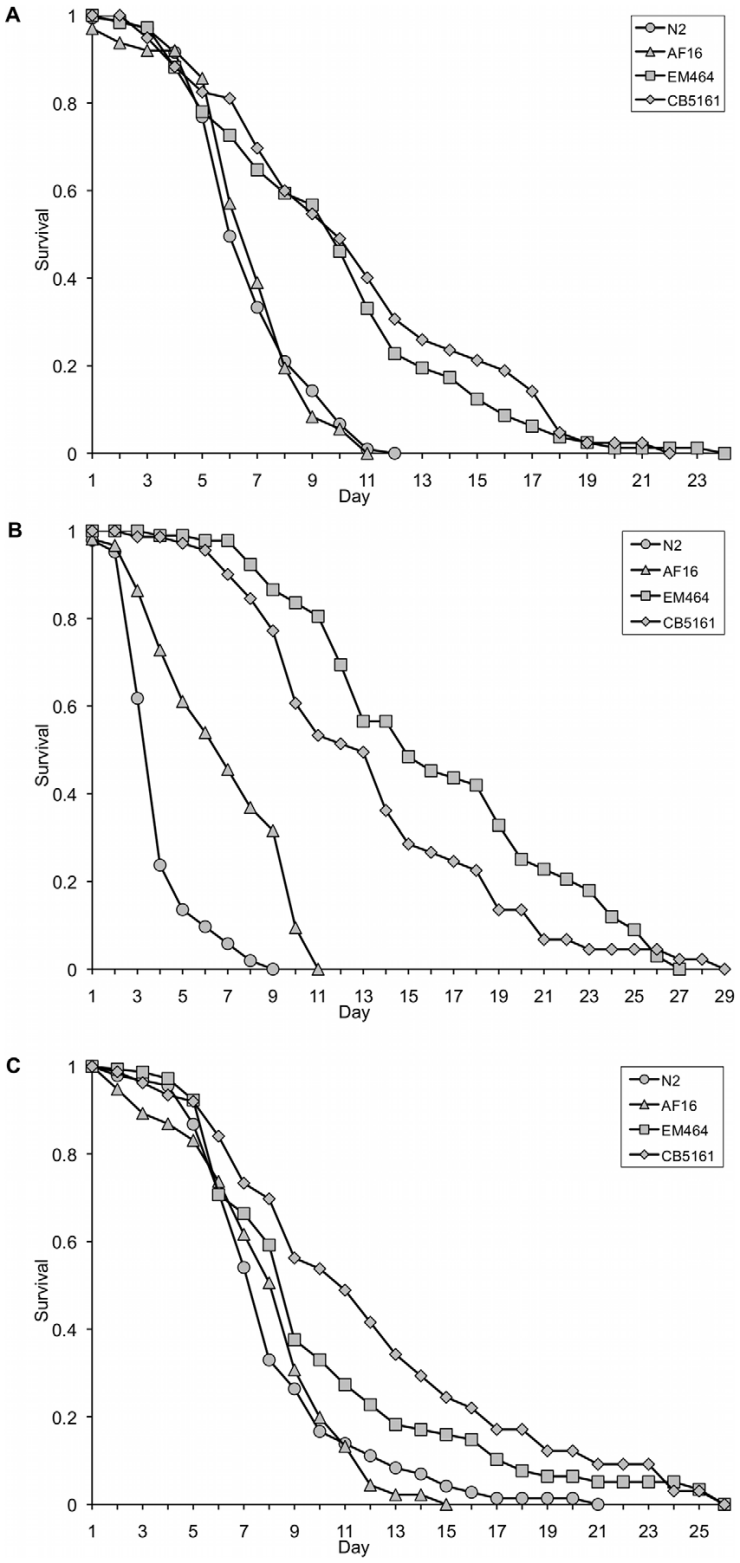
Survival analysis following exposure to biotic stress (three species of pathogenic bacteria). Survival of L4-stage hermaphrodites or female animals during exposure to pathogenic bacteria. *C. remanei* and C. *brenneri* are significantly more resistant to *Pseudomonas aeruginosa* (Figure 4a, p<0.0001, Table S1) and *Staphylococcus aureus* (Figure 4b, p<0.0001, Table S1). However, all four species show similar susceptibility to *Salmonella typhimurium* (SL1344) (Figure 4c, p>0.05, Table S1).

Numerous human pathogens are now known to be lethal towards *C. elegans* [25], [26], [27], [28], [29], [30], [31]. Since DAF-16 activity contributes towards stress resistance during infection [32], we assessed whether the four *Caenorhabditis* species varied in resistance to a range of pathogens. Interestingly, type strains of the two long-lived, gonochoristic species (EM464 and CB5161) showed significantly higher resistance to the Gram-negative bacterium *Pseudomonas aeruginosa* (Figure 4a), the Gram-positive bacterium *Staphylococcus aureus* (Figure 4b) and the fungus *Cryptococcus neoformans* [21] than type strains of the two hermaphroditic species (AF16 and N2). However, all four species showed similar sensitivity to the Gram-negative pathogen *Salmonella typhimurium* (Figure 4c). To ensure that these differences were not isolate dependent, we tested multiple additional isolates of each species for resistance to *S. aureus*. In all cases, gonochoristic isolates exhibit substantially higher resistance to killing by this pathogen (Table S2 and Figure S1), suggesting that the higher DAF-16 levels in the two gonochoristic species may potentially drive enhanced resistance to some, but not all, pathogens.

Since progeny production and the consequent risk of matricidal killing has previously been shown to shorten *C. elegans* lifespan, particularly when exposed to pathogens, [30], [33], [34] we considered the possibility that the enhanced survival of gonochoristic species may result from the absence of matricidal killing. To test this, we exposed feminised (and thus infertile when singled) *C. elegans* animals (BA17, fem-1(hc17)) to the pathogenic bacteria *S. aureus*. As previously reported, feminised *C. elegans* exhibited improved survival under pathogenic conditions (Figure S3), but this increase is nowhere as significant as the increase in lifespan seen in the higher *daf-16* producing gonochoristic species on *S. aureus*. Thus the enhanced survival of gonochoristic species is not attributable to the lack of progeny production.

### Manipulation of the DAF-16 pathway

**Figure 5:**
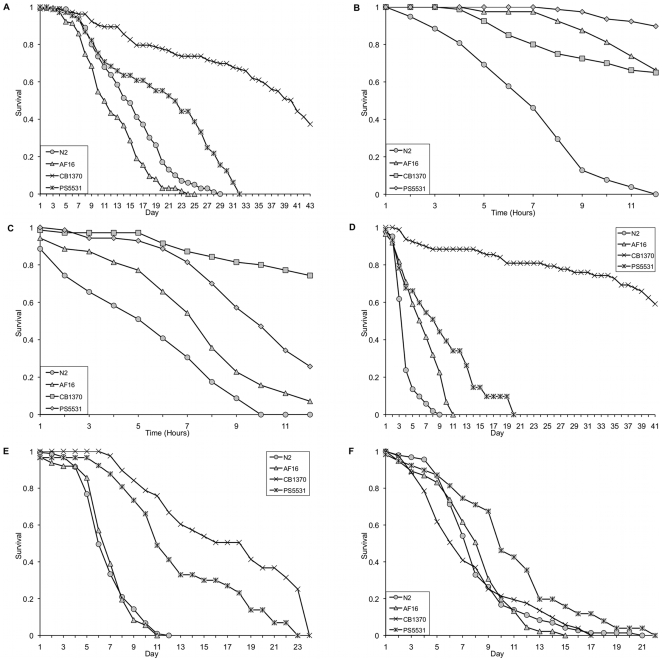
The effect of *daf-2* mutations on lifespan and resistance to abiotic and biotic stress. *daf-2* mutations in *C. elegans* (CB1370) and *C. briggsae* (PS5531) result in enhanced lifespan (Figure 5a) and resistance to high temperature (37°C, Figure 5b) or 7 mM copper chloride (Figure 5c). Both mutants also show significantly higher resistance to killing by *Staphylococcus aureus* (Figure 5d) and *Pseudomonas aeruginosa* (Figure 5e), although the magnitude of the resistance is significantly lower for *C. briggsae daf-2* than for the equivalent mutation in *C. elegans*. In contrast, the *daf-2* mutation does not enhance the resistance of either species to *Salmonella typhimurium* (Figure 5f).

*C. elegans* DAF-16 activity can be dramatically enhanced by loss-of-function mutations in the upstream insulin-like growth factor receptor DAF-2 [35], [36]. We investigated whether this phenomenon is conserved in *C. briggsae*, which, like *C. elegans*, has low basal levels of DAF-16 (Figure 2a), by comparing *daf-2* loss-of-function mutants in both species. As previously reported [7] we observed that *C. briggsae* (*daf-2*) mutants, have increased longevity relative to wildtype animals (Figure 5a). In addition, inactivation of *daf-2* in *C. briggsae* enhances resistance to high temperature (Figure 5b) and heavy metal toxicity (Figure 5c), as it does in *C. elegans* [10], [37]. Interestingly, *C. briggsae daf-2* mutants show enhanced resistance towards *S. aureus* (p<0.02, Table S4) and *P. aeruginosa* (p<0.0001, Table S4), but the magnitude of the increase is substantially smaller than that for *C. elegans daf-2* mutants (Figures 5d and 5e). Finally, loss of *daf-2* did not enhance resistance to *S. typhimuri*um in either *C. elegans* or *C. briggsae* (Figure 5f). Regrettably, genetic mutants in *daf-2* or *daf-16* are not available for either gonochoristic species, nor is RNA interference efficient enough in these species to allow direct manipulation of the IIS pathway in a similar manner. However, should such studies become feasible in the future, then our data would predict that loss of *daf-2* would likely have only a minimal effect on lifespan and stress resistance in the gonochoristic species.

### Comparative analysis of the DAF-16 regulon in *C. elegans, C. briggsae* and *C. remanei*

We considered the possibility that the DAF-16 pathway itself may have become modified during the diversification of the Caenorhabditid nematodes. However, calculation of Ka/Ks ratios for all of the components in the IIS signalling pathway (*daf-2*, *age-1*, *pdk-1*, *akt-1*, and *daf-16*) between *C. elegans*, *C. briggsae* and *C. remanei* showed no evidence for positive selection in any of the genes (Table S5).

Given that the IIS pathway itself does not appear to have been modified during the evolution of these species, we next investigated whether the downstream targets of DAF-16 differed between the three sequenced nematode species (*C. elegans*, *C. briggsae* and *C. remanei*). We searched for the presence of perfectly matched DAF-16 canonical consensus sites (ttatttac/gtaaataa, ttgtttac/gtaaacaa) in the 3kb upstream of every predicted gene in *C. elegans, C. briggsae* and *C. remanei*. In *C. elegans* our approach yielded 6293 genes (31.2% of the genome) containing either one or both of the known sites in their 3kb upstream region. In comparison, only 23.4% (5,150 genes) in *C. briggsae* and 26.7% (8,456 genes) in *C. remanei* contained at least one of the consensus sites. We note that the short length and relative variability of the DAF-16 consensus sequence means that this approach inherently overestimates the number of DAF-16 binding sites in the genome. However, given the absence of experimental techniques (such as chromatin immunoprecipitation) in the non-*elegans* species, such a bioinformatic approach is, at present, the only way of obtaining an approximate estimate of genome-wide differences in the IIS pathway within this group of organisms.

Based on this analysis, the number of orthologous genes that contain perfect matches to the DAF-16 consensus binding sites appears similar between *C. elegans* and *C. briggsae* (1900 genes), *C. elegans* and *C. remanei* (2111 genes) and *C. briggsae* and *C. remanei* (2165 genes). However, although *C. elegans, C. briggsae* and *C. remanei* have 13,015 genes in common (64.4% of the *C. elegans* genome) only 913 of these contain the DAF-16 binding elements in all three species, a group that we define as the core DAF-16 regulon (Figure S4).

Based on these gene sets, we asked whether the core DAF-16 regulon and the species-specific DAF-16 regulons differ in the type of genes they contain by testing whether particular gene ontology (GO) terms (using GOTERM BP_ALL and GOTERM BP_2) are overrepresented (Table S6 and Table S7). The DAF-16 core regulon shows, amongst others, enrichment for genes that are involved in lifespan regulation, immune response and responses to chemical stimuli (including detoxification and stress response) (Table S6). Intriguingly, whilst both the *C. elegans-*specific and *C. remanei-*specific DAF-16 regulons also show overrepresentation of genes involved in immunity (11 genes in *C. elegans*, 13 genes in *C. remanei*) and stress responses (38 genes in *C. elegans*, 14 genes in *C. remanei*) these groups are not overrepresented in the *C. briggsae*-specific DAF-16 regulon (Table S6).

**Figure 6:**
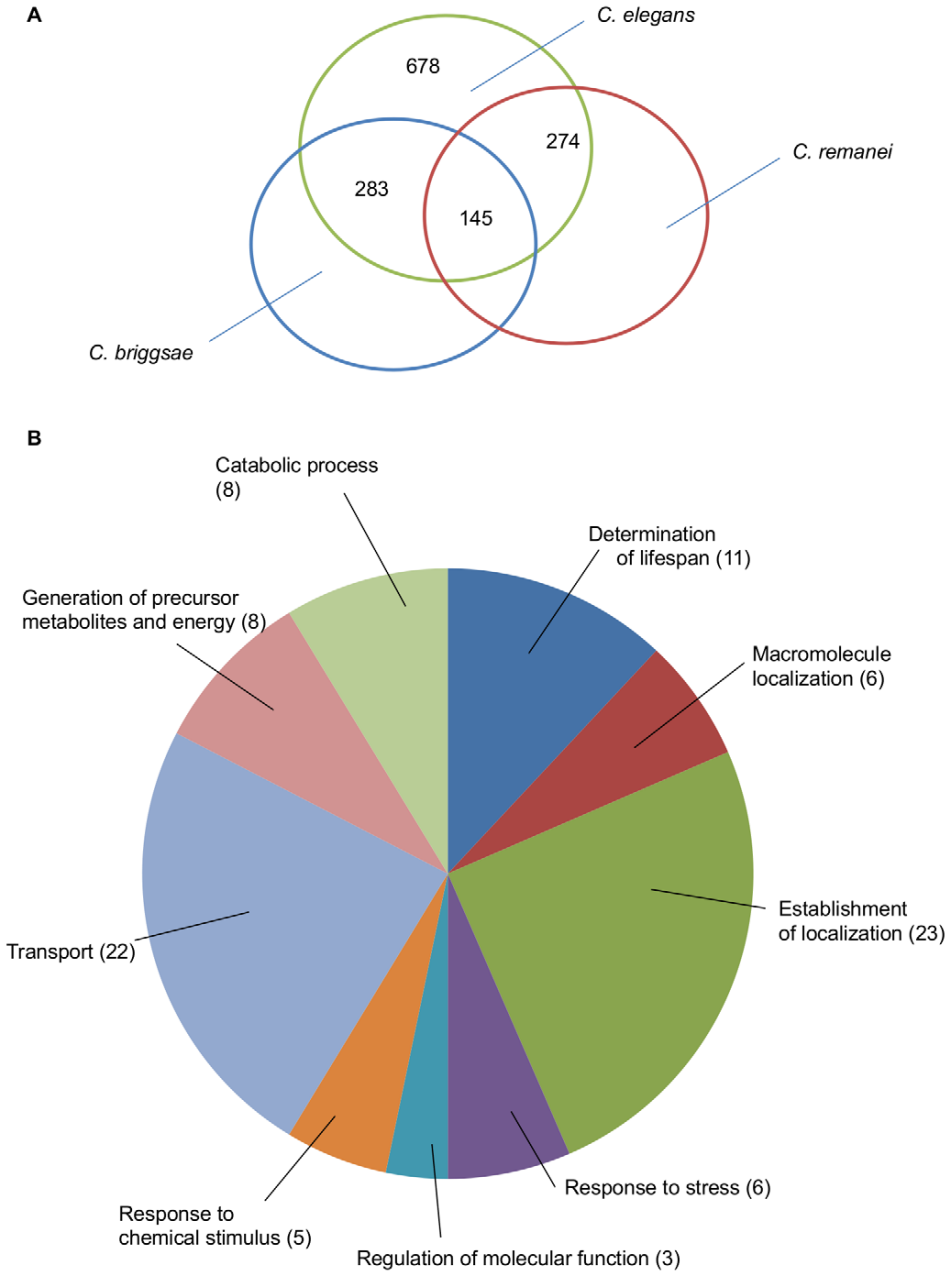
The DAF-16 regulon based on an adjusted *C. elegans* dataset. (a) Venn diagram of putative DAF-16 target genes in the three species. 145 orthologous genes have an upstream DAF-16 binding site in all three species, a group we define as the core DAF-16 regulon. (b) The genes within the core DAF-16 regulon were tested for over-representation of particular annotation categories within GOTERM BP_2 provided by the database DAVID. In brackets are the number of genes in the core DAF-16 regulon which are associated with a particular GO term within GOTERM BP_2.

In order to reduce the number of false positives in our *C. elegans* dataset we compared it to a gene list containing all putative DAF-16 targets recently identified in *C. elegans* via a range of other approaches by Oh [38], Murphy [14], Halaschek-Wiener [39], Lee [16], McElwee [40] and Dong [41]. Altogether, 1746 genes were identified as putative DAF-16 targets in at least one of these other datasets and 678 of these were also identified by our approach, a group we refer to as the adjusted dataset. Of the 678 potential *C. elegans* DAF-16 target genes, 283 overlap with the *C. brigssae* dataset and 274 genes were found in the *C. remanei* list. The adjusted DAF-16 core regulon (genes found in all three species) contains 145 genes (Figure 6a).

Partitioning the adjusted DAF-16 core regulon using the GOTERM BP_ALL and GOTERM BP_2 gene categories revealed significant enrichment for genes involved in the regulation of lifespan, stress response, transport, localization and metabolism (Figure 6b and Table S8). As expected the outcomes of the analyses of the unadjusted and the adjusted datasets differ slightly. However, the overall pattern is the same between the two approaches for both the core regulon as well as the species-specific regulon.

Finally, we compared the list of putative *C. elegans* DAF_16 targets identified by Oh and colleagues via a direct, chromatin immunoprecipitation (ChIP) approach [38] with those identified via microarray or bioinformatic approaches in the other studies (Murphy [14], Halaschek-Wiener [39], Lee [16], McElwee [40] and Dong [41]) or our own dataset. (Table 1). Interestingly, there is very little overlap between DAF-16 targets identified by ChIP and those inferred from microarray or bioinformatic analysis, with the exception of 11 genes shared between Oh et al and McElwee et al and 30 genes shared between Oh et al and our dataset. Thus there is likely to be considerable benefit in combining a range of experimental approaches in order to narrow down the list of true DAF-16 target genes.

**Table 1:** Overlap of the number of potential DAF-16 targets.

	Lee	Murphy	Wiener	McElwee	Dong	This study
**Overlapping with Oh**	1/81	2/473	1/317	11/953	1/93	30/6293

This table shows the overlap between the reference dataset of Oh and other datasets obtained by Murphy [14], Halaschek-Wiener [39], Lee [16], McElwee [40] and Dong [41] and the *C. elegans* gene list of this study. The first number gives the number of genes that are shared between Oh and the dataset of comparison. The second number stands for the total number of genes identified as potential downstream targets of DAF-16 in the corresponding study.

## Discussion

It is now clear that the lifespan of an organism is determined by a combination of environmental conditions, stochastic factors (such as lifestyle) and genetic background. Numerous studies have demonstrated that the evolutionarily conserved transcription factor DAF-16 is a critical gene regulator that controls the transcription of hundreds of genes involved in immunity, stress responses and longevity in *C. elegans* [42]. The homologues of *daf-16* in other organisms have been shown to perform similar functions [18] and yet species differ significantly in terms of lifespan and immunity, raising the question of how such DAF-16 mediated phenotypes have changed through evolutionary time.

Here we show covariance of three DAF-16 mediated phenotypes, longevity, immunity and stress response, across the *Caenorhabditis* genus. Strikingly, the two gonochoristic species (*C. remanei* and *C. brenneri*) show significantly higher basal expression of DAF-16 than the shorter-lived hermaphroditic species *C. elegans* and *C. briggsae*. Thus, enhanced expression and/or activation of DAF-16 may be an important mechanism by which species regulate a combination of phenotypes that enhance resistance to abiotic and biotic stresses and hence favour a longer life. The fact that this pattern is seen in multiple isolates of two gonochoristic species may reflect their need to search for a partner to mate, a lifestyle that increases the chance of encountering stressful conditions (eg. pathogens, high temperature) and is likely to favour the evolution of a longer lifespan in order to increase mating opportunities. In addition, since we know very little about the natural ecology of the *Caenorhabditis* nematodes [43], it is possible that differences in the niches inhabited by these species may impose extrinsic stresses that have led to the evolution of improved stress tolerance via the over-expression of DAF-16.

It is interesting to note that susceptibility to several pathogens correlates with other DAF-16 mediated effects, with the exception of the Gram-negative bacterium *S. typhimurium*, which shows similar lethality in all four species and the two *daf-2* mutants. Since *S. typhimurium* is one of the few human pathogens thus far shown to establish a truly persistent infection in the worm due to its resistance towards antimicrobial peptides [25], [44], this finding may indicate that DAF-16 plays little or no role in dealing with gut-colonising pathogens.

The insulin-like signalling pathway contributes to both innate immune responses and stress responses in *C. elegans*. Our data suggests that this may also hold true in closely related nematode species. In line with this, we show that the components of this pathway do not show evidence of adaptive sequence evolution during the diversification of these species whereas the complement of putative downstream targets controlled by DAF-16 appear to vary between these species. All three sequenced species share a core DAF-16 regulon comprised of genes functioning in longevity, stress response and other biological processes. However, whilst *C. elegans* and *C. remanei* contain a similar set of target types in their species-specific DAF-16 regulons, the species-specific DAF-16 regulon of *C. briggsae* lacks genes involved in immunity and stress response. Interestingly, in line with this finding, we observed that a *daf-2* mutant in *C. briggsae* is long-lived and resistant to abiotic stress, but only moderately resistant to killing by a range of pathogens.

The majority of enriched genes identified by our approach are associated with other biological processes such as metabolism, transport and other functions, in line with previous studies that have identified downstream targets of DAF-16 in *C. elegans* [14], [38], [39], [40], [45], [46]. We note, however, that such bioinformatic analyses are susceptible to false positive (due to the chance occurrence of DAF-16 consensus sequences) and false negative (due to its reliance on perfect-match sequence motifs) errors. Indeed, depending on the approach used, others have estimated that up to 78% of *C. elegans* genes might be potential DAF-16 downstream targets [42]. As such, we would emphasize that our bioinformatic analysis is intended only as a guide for future experimental analyses once tools become available.

In conclusion, we demonstrate covariance of DAF-16 mediated phenotypes in the four most well-characterized species of the *Caenorhabditis* clade. We note that our data are correlative but, as yet, cannot prove a causative influence of *daf-16* expression level on these phenotypes in the gonochoristic species. Currently, demonstrating a direct role for DAF-16 in phenotypic covariance in the gonochoristic nematodes is not technically feasible. Very few genetic mutants have been made in these species, RNA interference is of low efficiency and no antibodies exist for chromatin immunoprecipitation approaches. However, many groups are currently attempting to develop such tools for these species and, as such, we hope that a full mechanistic investigation of the IIS pathway in non-elegans species will be feasible within the next few years.

## Materials and Methods

### Bacterial strains and growth conditions

*Escherichia coli* OP50 [47], *Salmonella typhimurium* SL1344 [48], *Pseudomonas aeruginosa* PA01 and *Staphylococcus aureus* NCTC8532 were grown in nutrient rich Luria-Bertani (LB) broth overnight with shaking at 37°. The bacterial culture was then seeded onto standard Nematode Growth agar Medium (NGM[47], [49]) plates. These plates were incubated overnight at 37° (∼16 hrs) followed by storage at 4°. Plates were always equilibrated to room temperature before use.

### Worm Strains

Worm strains N2, RC301, CB4856 (wildtype *Caenorhabditis elegans*), AF16, ED3033, ED3034 (wildtype *Caenorhabditis briggsae*), EM464, JU1082, JU1084 (wildtype *Caenorhabditis remanei*), CB5161, LKC28, SB129 (wildtype *Caenorhabditis brenneri*), CB1370 [*C. elegans daf-2*(e1370)], PS5531 [*C. briggsae daf-2*(sy5445)] and BA17 [*C.elegans fem-1* (hc17)] were grown on standard NGM plates seeded with OP50 strain of *E. coli* bacteria as a food source [47], [49]. All strains except BA17 (which was grown at 25°C to induce feminisation) were grown at 20°C. Fourth larval stage hermaphrodites from the hermaphroditic species and females from the gonochoristic species were used for all the phenotypic assays performed.

### Longevity/Pathogen Assays

The hermaphroditic and gonochoristic strains of worms were bleached [49] to produce age-synchronous L4 molt populations. Between 80 and 250 L4 worms from the hermaphroditic species, or females in the case of gonochoristic species, were transferred onto NGM plates (∼30 worms per plate, yielding up to 10 replicates) seeded with OP50 for longevity assays and SL1344, PA01 and NCTC8532 for pathogen assays as food source. Plates with OP50 were incubated at 20°C with the rest being incubated at 25°C. Worms grown at 25°C on OP50 have been shown to have a significantly longer lifespan than those grown on pathogenic bacteria such as *S. typhimurium* [25] which eliminates the negative effects of heat as an experimental determinant. The worms on all these plates were scored for survival every 24 hrs. Animals were considered dead when they failed to respond to prodding by a platinum wire. The worms were transferred onto new plates every one to two days until they stopped egg laying, in order to prevent F1 progeny from interfering with the experiment.

### Heat Shock Assay

L4 worms from the hermaphroditic species, and females in the case of gonochoristic species, were transferred onto NGM plates with OP50 that had been prewarmed to 37°. These plates were then incubated at 37°C and the worms were scored for survival at hourly intervals.

### Metallotolerance Assay

Age synchronous L4 worms were transferred from NGM plates into 24-well tissue culture plates containing copper chloride (7 mM) dissolved in K medium (53 mM NaCl, 32 mM KCl) [10], [50]. The plate was incubated at 20°C and the worms were scored for survival every hour.

### Statistical Analysis

Survival curves were produced based on the Kaplan-Meier method using MS-Excel and the significance was calculated using the non-parametric log-rank method. Assays were then corrected for multiple testing using the Bonferroni correction.

### Preparation of total nematode mRNA and Quantitative RT PCR

For qRT-PCR, RNA was isolated from a mixed larval stage population of each of the four species of worms. These worms were grown on NGM plates with OP50 as food source at 20°. Animals were then washed off the plates using M9 buffer, followed by repeated washes again with M9 buffer before being homogenized using the Precellys 24 machine (Stretton Scientific). The RNA was then isolated from these worm samples using the Qiagen RNeasy Mini kit (cat. No. 74140) using the manufacturer's protocol. RNA was then reverse transcribed into cDNA using Superscript II reverse transcriptase (Invitrogen) according to the manufacturer's instructions. Real-time quantitative RT-PCR was performed (7300 Real Time PCR System; Applied Bio Systems) on this cDNA using the SYBR Green PCR kit (Quantace) to determine the expression levels of *daf-16* across the four species. Primers for this were designed manually and tested for maximum efficiency with their respective cDNA prior to qRT-PCR. Primers used include *daf-16* primers for *C. elegans, C. briggsae, C. remanei* and *C. brenneri* (Table 2).

**Table 2:** List and sequence of primers used for studying daf-16 expression levels using Real Time PCR.

Gene	Species	Forward Primer 5′	Reverse Primer 3′
*daf-16*	*C. elegans*	GCGAATCGGTTCCAGCAATTCCAA	ATCCACGGACACTGTTCAACTCGT
*daf-16*	*C. briggsae*	AGAAGGCTACCACTAGAACCAACG	TCCATCCAGCGGAACTGTTCGAAT
*daf-16*	*C. remanei*	CGACGGCAATACTCATGTCAATGG	ACGGTTTGAAGTTGGTGCTTGGCA
*daf-16*	*C. brenneri*	CCTTAGTAGTGGCCTCAATGGTGT	CACAACCTATCACTTCACTCTCGC
*gpd-3*	All species	TGAAGGGAATTCTCGCTTACACC	GAGTATCCGAACTCGTTATCGTAC

The RT PCR levels were normalized to the housekeeping gene, Glyceraldehyde 3-Phosphate Dehydrogenase (*gpd*-3). The primers for this gene were; Primer Fwd – TGAAGGGAATTCTCGCTTACACC and Primer Rev – GAGTATCCGAACTCGTTATCGTAC. We confirmed that our results were not due to variation in *gpd-3* by cross checking RT PCR levels against another reference gene, 18sRNA, the primers for which were; Primer Fwd - TTCTTCCATGTCCGGGATAG and Primer Rev – CCCCACTCTTCTCGAATCAG. To assess the efficacy of the primers and the sensitivity of the qPCR assay, 2-fold dilution series of the template DNA for all the species tested were prepared and subjected to qPCR amplification. The results obtained were extrapolated to produce standard curves by linear regression analysis between threshold cycle (Ct) and sample dilution that gave coefficients of determination (r^2^) that exceeded 0.95 for all template/primer combinations (Table S9). Once amplification efficiencies of the target and the reference were determined to be approximately equal, RT PCRs were carried out for all the experimental conditions. These results were analysed using the ΔC_T_ method with the *gpd-3* and 18S RNA levels as controls for normalization and expressed as fold change compared to *C. elegans* [51].

### Bioinformatic analysis of DAF-16 downstream targets

The complete genomes of *C. elegans* (20,189 genes), *C. briggsae* (21,976 genes) and *C. remanei* (31,614 genes) were downloaded from Wormbase release WS 197 (www.wormbase.org). We surveyed a 3000 bp upstream flanking region of each gene (upstream of the lead ATG) for the presence of the two known canonical DAF-16 binding sites (ttatttac/gtaaataa, ttgtttac/gtaaacaa; [52]). We applied a perfect match approach using the dna-pattern tool implemented in the freely available software package Regulatory Sequence Analysis Tools (RSAT; http://rsat.bigre.ulb.ac.be/rsat/; [53]). Only genes with upstream flanking region containing one or more perfect matches to the consensus sites were included in further analyses. From this set of genes we then retrieved a subset of genes for each species that are orthologous either between *C. elegans* and *C. briggsae, C. elegans* and *C. remanei* or between all three species. Based on these orthologous gene sets we defined the following classes: i) a species-specific DAF-16-regulon, consisting of orthologs that contain the consensus site in only one of the species, ii) the species-shared DAF-16 regulon, consisting of orthologous genes that contain the consensus site in two of the species and iii) the core-DAF-16 regulon, consisting of orthologs that contain the consensus site in all three species. These gene subsets were subsequently analysed in order to identify enriched functional gene groups. This analysis was performed using the functional annotation tools available from the non-commercial bioinformatic database DAVID (Database for Annotation, Visualization and Integrated Discovery) [54], [55].

Furthermore we compared the resulting *C. elegans* gene list to the available datasets of Oh [38], Murphy [14], Halaschek-Wiener [39], Lee [16], McElwee [40] and Dong [41]. These datasets were combined, duplicates removed and subsequently run against the *C. elegans* gene list (containing all genes with a perfect match to one of the binding motifs). The resulting gene list was then compared to the lists obtained for *C. briggsae* and for *C. remanei*. The three resulting lists were analysed using the functional annotation tools in DAVID. Finally, we looked whether there was any overlap between the dataset of Oh and the datasets of Murphy, McElwee, Lee, Halaschek-Wiener, Dong.

We determined the number of genes that were enriched within the functional annotation category Gene Ontology GOTERM BP_ALL and especially enriched in GOTERM BP_2. The results were obtained by using the Functional Annotation Chart tool. The GOTERMS BP are available from the DAVID database. The p-value obtained in this analysis is equivalent to the EASE score, which uses a conservative adjustment of the Fisher's exact probability, and was applied to identify significantly enriched gene categories. DAVID provides several methods to correct for multiple testing which include Bonferroni adjustment of the p-value, and the Benjamini-Hochberg approach to control for family-wide false positive rate. The fold enrichment value measures the magnitude of enrichment and is considered significant if 1.5 or above [54]. For more statistical details and detailed description of the annotation methods used in DAVID please refer to the cited references above and references therein.

For all orthologs, the corresponding WormBase IDs of *C. elegans* genes were used as input files. Orthologs between *C. remanei* and *C. briggsae* but not occurring in *C. elegans* could not be addressed with this approach. All orthologous genes with a duplicate output in one of the species were counted only as one gene.

### Adaptive sequence evolution

Adaptive sequence evolution along the IIS pathway was studied in *C. elegans*, *C. briggsae* and *C. remanei*. Protein sequences and DNA sequences of the coding regions ranging from *daf-2*, *age-1*, *pdk-1*, *akt-1*, and *daf-16* were obtained from WormBase WS197 (www.wormbase.org). Protein sequences and DNA coding regions were aligned using ClustalX2 [56]. For each gene of interest, the presence of adaptive sequence evolution (ratio between synonymous [K_S_] and non-synonymous [K_A_] substitutions) was calculated between a pair of sequences (*C. elegans* and *C. brigssae*; *C. elegans* and *C. remanei*; *C. briggsae* and *C. remanei*) using PAL2NAL [57]. PAL2NAL calculates K_S_ and K_A_ by the codeml program in PAML. Briefly, pairwise protein alignments in CLUSTAL format and the corresponding DNA sequence alignments in FASTA format were used as input files. The following option settings were used. (i) Codon table: “universal”. (ii) Remove gaps and inframe stop codons: “Yes”. iii) Calculate K_S_ and K_A_: “Yes”. (iv) Remove mismatches: “No”.

## Supporting Information

Figure S1 Lifespan analysis of three wild isolates for each of the type strains of the tested *Caenorhabditis* species under pathogenic (*Staphylococcus aureus* NCTC8532) stress.
(0.91 MB TIF)

Figure S2 Lifespan analysis of three wildtype isolates for each of the type strains of the tested *Caenorhabditis* species under heavy metal stress.
(0.85 MB TIF)

Figure S3 Lifespan analysis of *C. elegans* feminizing mutant (BA17, *fem-1*(hc17)), *C. elegans* wild type (N2) and *C. remanei* (EM464) under pathogenic (*Staphylococcus aureus* NCTC8532) stress.
(0.87 MB TIF)

Figure S4 Figure illustrating the 913 genes of the 13,015 genes shared in common between *C. elegans, C. briggsae* and *C. remanei* that contain DAF-16 binding sites which we define as the core DAF-16 regulon.
(0.64 MB TIF)

Table S1 Complete list of the p values between all the type strains for the various assays performed. P values in bold indicate significance after testing for multiplicity using the Bonferroni correction.
(0.02 MB XLS)

Table S2 List of p values for all the wildtpe isolates compared to the type *C. elegans* strain (N2) for the assays performed. P values in bold indicate significance after testing for multiplicity using the Bonferroni correction.
(0.02 MB XLS)

Table S3 Table represents the fold change in expression levels of *daf-16* among the tested *Caenorhabditis* species, including *C. elegans* males.
(0.02 MB XLS)

Table S4 Complete list of the p values between type strains (N2 and AF16) and their *daf-2* mutant counterparts (CB1370 and PS5531) for the various assays performed. P values in bold indicate significance after testing for multiplicity using the Bonferroni correction.
(0.01 MB XLS)

Table S5 Adaptive sequence evolution of the IIS pathway in *C. elegans, C. remanei* and *C. briggsae*.
(0.03 MB XLS)

Table S6 Table represents the outcome of the GOTERM BP_2 DAVID analysis performed for the DAF-16 core regulon and the species specific DAF-16 regulon. The gene count gives the number of genes within the tested gene sets which are found to be associated with a GO group within GOTERM BP_2. P-value is based on a conservative adjustment of the Fisher's exact probability and was subsequently coorected for multiple testing with Bonferroni correction and the Benjamin-Hochberg method implemented in DAVID. After correction the p-value gets larger. The fold enrichment value measures the magnitude of enrichment.
(0.08 MB XLS)

Table S7 Table represents the outcome of the GOTERM BP_ALL DAVID analysis performed for the DAF-16 core regulon and the species specific DAF-16 regulon. The gene count gives the number of genes within the tested gene sets which are found to be associated with a GO group within GOTERM BP_ALL. P-value is based on a conservative adjustment of the Fisher's exact probability and was subsequently coorected for multiple testing with Bonferroni correction and the Benjamin-Hochberg method implemented in DAVID. After correction the p-value gets larger. The fold enrichment value measures the magnitude of enrichment.
(0.20 MB XLS)

Table S8 Table representing the adjusted DAF-16 core regulon using the GOTERM BP_ALL and GOTERM BP_2 gene categories which revealed significant enrichment for genes involved in the regulation of lifespan, stress response, transport, localization and metabolism.
(0.13 MB XLS)

Table S9 Table represents the coefficients of determination (r^2^) for all template/primer combinations. Once amplification efficiencies of the target and the reference genes were determined to be approximately equal, RT PCRs were carried out for all the experimental conditions.
(0.02 MB XLS)
