# Supplementary figures and images for: Phenotypic Covariance of Longevity, Immunity and Stress Resistance in the Caenorhabditis Nematodes
Source: PLoS One. 2010 Apr 1;5(4):e9978. doi: 10.1371/journal.pone.0009978 (PMC2848519; doi:10.1371/journal.pone.0009978)

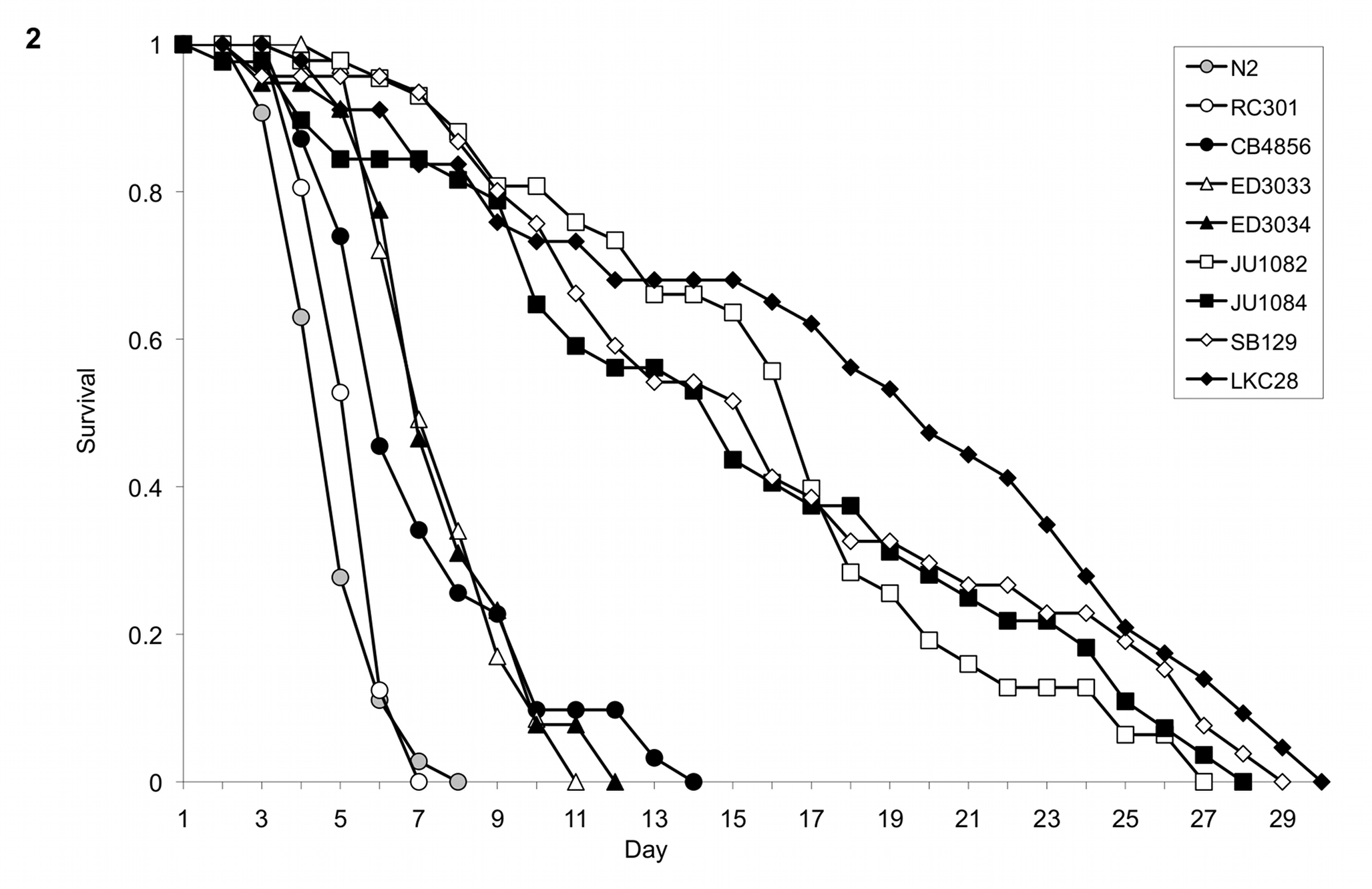

Supplement: Figure S1 — Lifespan analysis of three wild isolates for each of the type strains of the tested Caenorhabditis species under pathogenic (Staphylococcus aureus NCTC8532) stress. (0.91 MB TIF) [file pone.0009978.s001.tif]

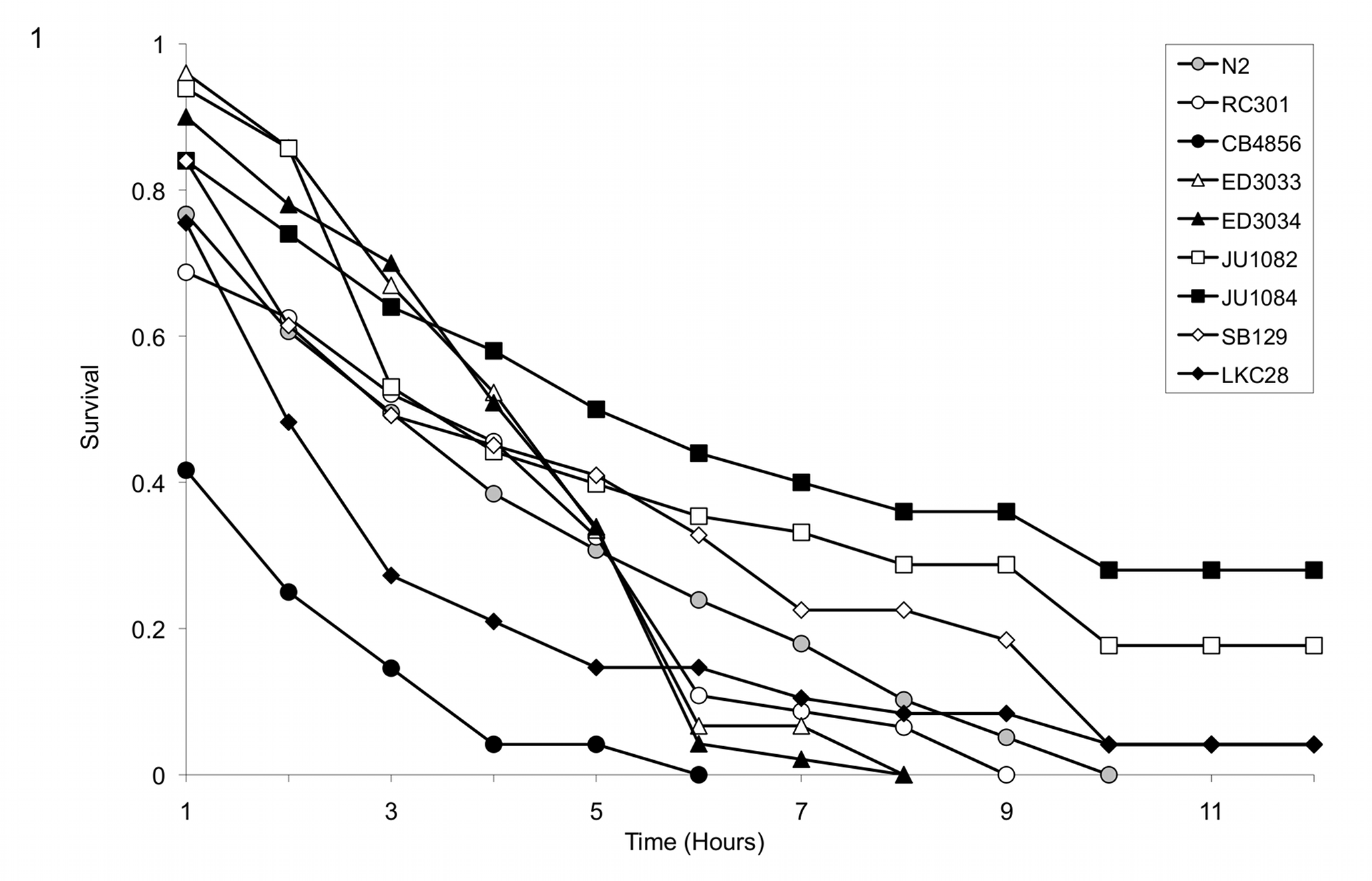

Supplement: Figure S2 — Lifespan analysis of three wildtype isolates for each of the type strains of the tested Caenorhabditis species under heavy metal stress. (0.85 MB TIF) [file pone.0009978.s002.tif]

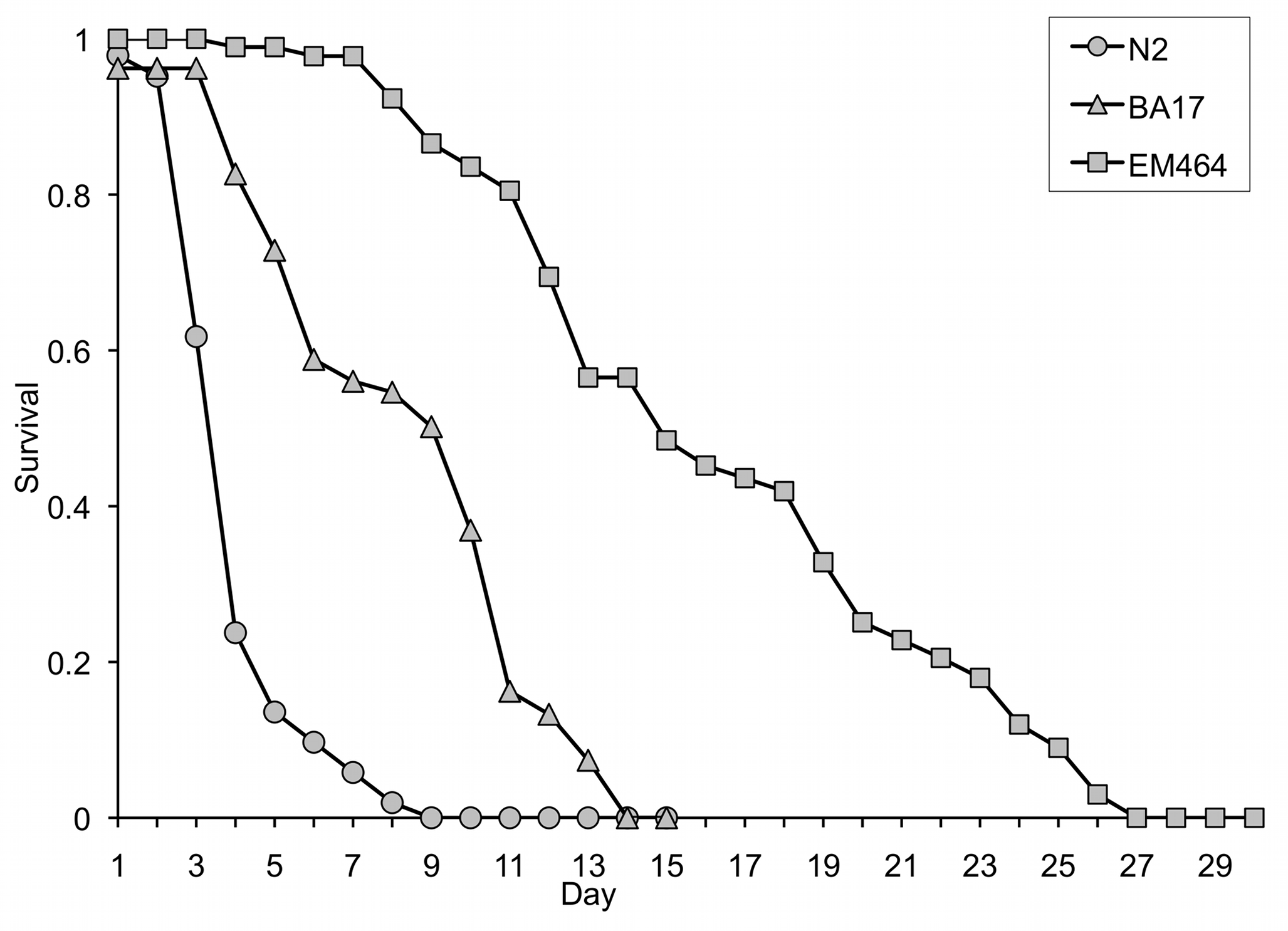

Supplement: Figure S3 — Lifespan analysis of C. elegans feminizing mutant (BA17, fem-1(hc17)), C. elegans wild type (N2) and C. remanei (EM464) under pathogenic (Staphylococcus aureus NCTC8532) stress. (0.87 MB TIF) [file pone.0009978.s003.tif]

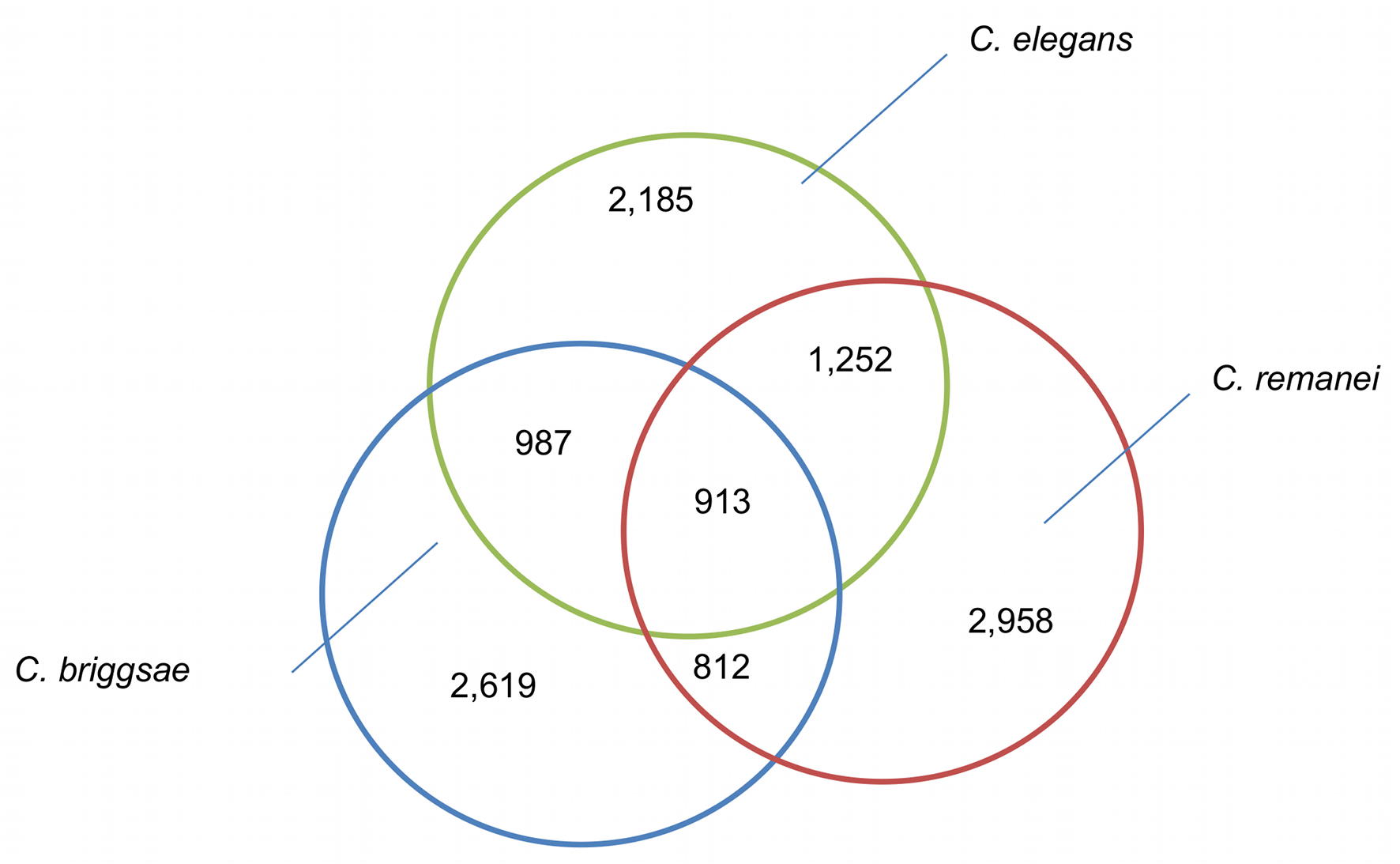

Supplement: Figure S4 — Figure illustrating the 913 genes of the 13,015 genes shared in common between C. elegans, C. briggsae and C. remanei that contain DAF-16 binding sites which we define as the core DAF-16 regulon. (0.64 MB TIF) [file pone.0009978.s004.tif]
